# Mitochondrial genome of an Allegheny Woodrat (*Neotoma magister*)

**DOI:** 10.1080/23802359.2018.1437806

**Published:** 2018-02-22

**Authors:** Mandy Schofield, Joseph Duchamp, Jeffery L. Larkin, Timothy J. Smyser, Jacqueline M. Doyle

**Affiliations:** a Department of Biological Sciences, Towson University, Towson, MD, USA;; b Department of Biology, Indiana University of Pennsylvania, Indiana, PA, USA;; c USDA-APHIS-WS National Wildlife Research Center, Fort Collins, CO, USA;; d Department of Forestry and Natural Resources, Purdue University, West Lafayette, IN, USA

**Keywords:** Neotomini, cricitidae, peromyscini, cricetid

## Abstract

The Allegheny woodrat (*Neotoma magister*) is endemic to the eastern United States. Population numbers have decreased rapidly over the last four decades due to habitat fragmentation, disease-related mortality, genetic isolation and inbreeding depression; however, effective management is hampered by limited genetic resources. To begin addressing this need, we sequenced and assembled the entire Allegheny woodrat mitochondrial genome. The genome assembly is 16,310 base pairs in length, with an overall base composition of 34% adenine, 27% thymine, 26% cytosine and 13% guanine. This resource will facilitate our understanding of woodrat population genetics and behavioral ecology.

The Allegheny woodrat (*Neotoma magister*) is endemic to eastern North America, where population numbers have decreased rapidly since the 1980s (Wright 2008). Causes of the decline include habitat destruction, decreased food availability, increased mortality caused by *Baylisascaris procyonis* infection, genetic isolation and inbreeding depression (LoGiudice 2008; Smyser et al. 2012). Despite this threatened status, genetic studies have been limited (Castleberry et al. 2000, 2002; Smyser et al. 2012) and NCBI cites just 59 archived *N*. *magister* nucleotide sequences. In contrast, 369 and 1567 nucleotide sequences have been archived for the dusky-footed woodrat (*N. fuscipes*) and the Mexican woodrat (*N. mexicana*), respectively. Assembling the complete *N. magister* mitogenome will provide important resources for species identification (e.g., COI sequence) and contribute to our understanding of population structure across the species range.

We assembled the mitogenome of a single *N. magister* individual sampled in Westmoreland County, Pennsylvania (latitude/longitude (40.3, −79.3)). The sample is stored at −80 °C at Towson University in Baltimore County, Maryland (accession number J5). DNA was extracted with commercially available extraction (DNEasy Blood and Tissue, Qiagen, Venlo, the Netherlands) and clean-up (DNA Clean & Concentrator, Zymo Research, Irvine, California) kits, according to the instructions from the manufacturers. We conducted one lane of paired-end sequencing using an Illumina HiSeq2000 and used Trimmomatic (Bolger et al. 2014) to remove adaptors, discard short reads (<30 bp), and trim poor quality bases (Illumina Q-value ≤20) from both 5′ and 3′ ends of raw sequence reads.

We used a 12S rRNA sequence (accession number DQ179706.1) to seed mitogenome assembly implemented by MITObim and MIRA (baiting-and-iterative mapping; Chevreux et al. 1999; Hahn et al. 2013). The draft assembly was annotated using MITOS (Bernt et al. 2013). We used CLUSTALW implemented by MEGA 7.0.21 (Tamura et al. 2007) to align mitogenomes from *N. magister*, 19 other Cricetid species and an outgroup (*Sciurus vulgaris*) . This alignment was used to produce a maximum-likelihood tree using the GTR + G + I model of evolution and 1000 bootstraps (Figure 1).

**Figure 1.**
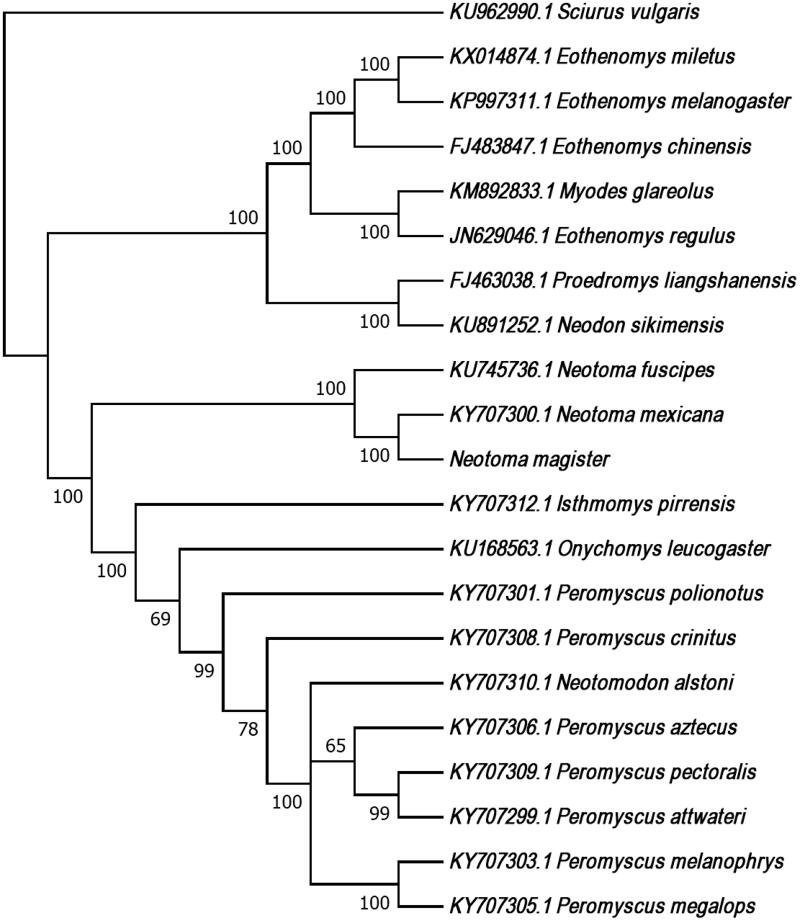
We used CLUSTALW implemented by MEGA 7.0.21 to align the mtDNA genome sequences from *N. magister*, *N. fuscipes*, *N. mexicana* (the only other woodrats for which complete mitogenome sequences were available from NCBI), 17 other Cricetid species and an outgroup (*Sciurus vulgaris*). This alignment was used to produce a maximum likelihood tree using the GTR + G + I model of evolution and 1000 bootstraps. Bootstrap values are included at each node and each species label includes the GenBank accession number, with the exception of *N. magister* (GenBank accession number MG182016).

The *N. magister* mitogenome (GenBank accession number MG182016) is 16,310 bp in length with 22 tRNAs, 2 rRNA genes and 13 protein-coding genes. Overall base composition includes 34% adenine, 27% thymine, 26% cytosine and 13% guanine. Gene order and organization are identical to that of *N. fuscipes* (Brown and Blois 2016) and *N. mexicana* (Sullivan et al. 2017). Our phylogeny indicates that *N. magister* is more closely related to *N. mexicana* than to *N. fuscipes*, a result consistent with a phylogeny generated using a combination of mitochondrial and nuclear markers (Matocq et al. 2007). Furthermore, as in Reeder et al. (2006), the clade including *Neotoma* species is sister to the clade including *Neotomodon*, *Onychomys*, and *Peromyscus* (i.e., Peromyscini).

This new genetic resource has important management implications. For example, our annotation of conserved mitochondrial genes will aid species identification from noninvasively collected samples. In addition, this mitogenome assembly may facilitate haplotyping across the *N. magister* range (e.g., by improving primer design), expanding upon our understanding of population structure and allowing us to identify evolutionarily significant units.
